# Cancer-Related Intracellular Signalling Pathways Activated by DOXorubicin/Cyclodextrin-Graphene-Based Nanomaterials

**DOI:** 10.3390/biom12010063

**Published:** 2022-01-01

**Authors:** Rosamaria Pennisi, Maria Musarra-Pizzo, Tania Velletri, Antonino Mazzaglia, Giulia Neri, Angela Scala, Anna Piperno, Maria Teresa Sciortino

**Affiliations:** 1 Department of Chemical, Biological, Pharmaceutical and Environmental Sciences, University of Messina, 98166 Messina, Italy; mmusarrapizzo@unime.it (M.M.-P.); giulia.neri@unime.it (G.N.); ascala@unime.it (A.S.); apiperno@unime.it (A.P.); 2 IFOM-Cogentech Società Benefit srl; via Adamello 16, 20139 Milan, Italy; tania.velletri@cogentech.it; 3 Istituto per lo Studio dei Materiali Nanostrutturati, Consiglio Nazionale delle Ricerche (ISMN-CNR), V.le F. Stagno d’Alcontres 31, 98166 Messina, Italy; antonino.mazzaglia@cnr.it

**Keywords:** graphene-based platform, cancer therapy, intracellular signalling pathway, doxorubicin

## Abstract

In the last decade, nanotechnological progress has generated new opportunities to improve the safety and efficacy of conventional anticancer therapies. Compared with other carriers, graphene nanoplatforms possess numerous tunable functionalities for the loading of multiple bioactive compounds, although their biocompatibility is still a debated concern. Recently, we have investigated the modulation of genes involved in cancer-associated canonical pathways induced by graphene engineered with cyclodextrins (GCD). Here, we investigated the GCD impact on cells safety, the HEp-2 responsiveness to Doxorubicin (DOX) and the cancer-related intracellular signalling pathways modulated by over time exposure to DOX loaded on GCD (GCD@DOX). Our studies evidenced that both DOX and GCD@DOX induced p53 and p21 signalling resulting in G_0_/G_1_ cell cycle arrest. A genotoxic behaviour of DOX was reported via detection of CDK (T14/Y15) activation and reduction of Wee-1 expression. Similarly, we found a cleavage of PARP by DOX within 72 h of exposure. Conversely, GCD@DOX induced a late cleavage of PARP, which could be indicative of less toxic effect due to controlled release of the drug from the GCD nanocarrier. Finally, the induction of the autophagy process supports the potential recycling of DOX with the consequent limitation of its toxic effects. Together, these findings demonstrate that GCD@DOX is a biocompatible drug delivery system able to evade chemoresistance and doxorubicin toxicity.

## 1. Introduction

**Scheme 1:**
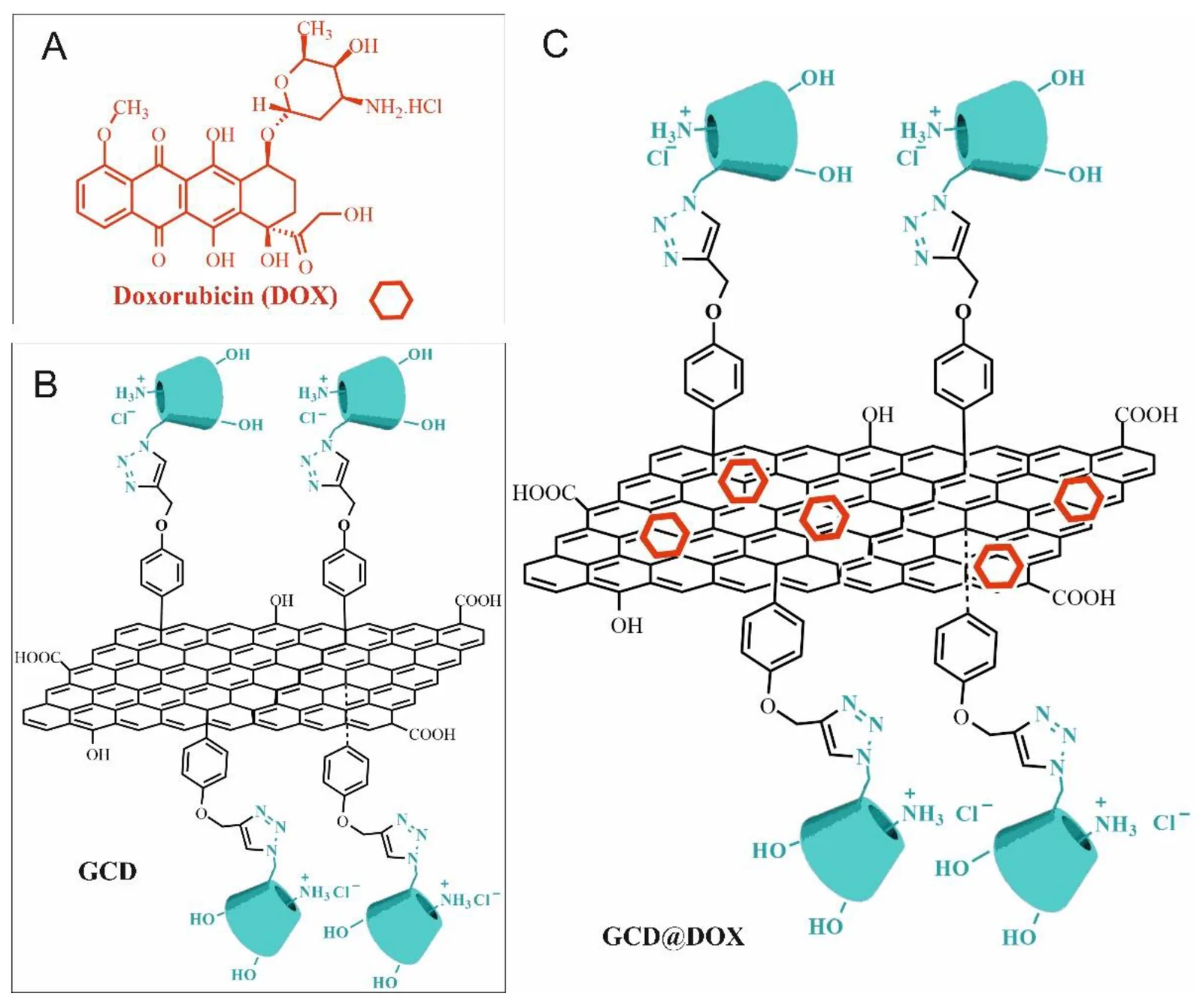
(**A**) Doxorubicin (DOX); (**B**) schematic sketch of graphene functionalised with cationic cyclodextrins (GCD platform); (**C**) schematic sketch of DOX loaded on GCD (GCD@DOX). (Reprinted from reference [18]).

Over the past three decades, the application of nanotechnology in medicine has had a significant impact on cancer disease treatment. Several nanotherapeutics have been clinically approved to support conventional anticancer therapies, while many others are under clinical investigation [1,2,3]. These nanoparticles are characterised by sizes ranging from a few to a few hundreds of nanometres (1–500 nm) and superior surface properties that are able to improve the delivery and release of therapeutic agents at the site of action [4]. However, to date, the most important challenge in nanomedicine remains reducing toxicity profile and enhancing therapeutic efficacy. Carbon-based nanomaterials, including nanodiamonds, fullerenes, carbon nanotubes, graphene, and carbon nanofibers, have been proposed as useful biological platforms due to their versatility, physical properties, and unique intracellular trafficking properties [5,6,7,8,9,10]. Surface functionalisation with polymers such as polyethylenimine (PEI, branched/linear), cationic dendrimers, glycopolymers, and poly amidoamine increases their solubility and intracellular uptake and stabilises their half-life in the cellular environment [11,12,13,14]. The engineering of graphene surfaces with macrocycles such as β-cyclodextrins (CD) allows the formation of stable inclusion complexes with hydrophobic small molecules by means of host–guest interactions with the hydrophobic CD cavity [15,16]. Recently, we reported a graphene nanoplatform covalently modified with functional cationic cyclodextrins (GCD) [17] as a carrier for the purposes of gene and drug delivery. We analysed its endocytosis internalisation mechanism, its perinuclear localisation, and its ability to deliver miRNAs. Specifically, we studied the effects of the GCD/miRNA15a complex on the regulation of the oncogene protein BCL2. The GCD platform was proved to be a promising therapeutic miRNA delivery system that was able to overcome the problems typically encountered, such as low stability, nuclease susceptibility and weak cellular uptake. Next, we investigated the intracellular fate of DOX loaded on GCD (GCD@DOX), including the nuclear distribution responsible for the gene expression modulation (Scheme 1) [18]. The GCD nanoplatform contains several molecular recognition sites (i.e., cationic external CD rims to provide electrostatic interactions; π systems to give π-π interactions and/or hydrophobic effects; CD hydrophobic cavities to provide host/guest inclusion complexes). According to literature data, DOX can interact with GCD by: (i) π-π stacking between the G conjugated structure and the quinone portion of DOX, as well as the hydrophobic effect between them; (ii) inclusion into the CD cavities; (iii) hydrogen bonding among OH and COOH groups of G and OH and NH2 groups of DOX [19,20]. Moreover, high formation constants were reported for inclusion complexes between functionalized βCDs and DOX in mixed water: DMSO solutions (2.3 × 10 ^4^ and 3.2 × 10 ^5^ M^−1^) [21]. A stronger interaction is expected in GCD with respect to the literature due to the GCD restored sp^2^ network and additional cationic sites on external CD rims. However, in our studies, GCD@DOX was prepared at a low actual loading (2.5%) [18] to avoid the overloading of DOX on the sp^2^ network [22], and the aspects related to the binding constant between DOX and GCD were not investigated. Caveolae-mediated endocytosis was detected as the main pathway of GCD cellular internalisation. FLIM and Raman mapping analyses indicated the presence of GCD only in the cytoplasm, whereas fluorescence microscopy showed the DOX release in the nuclear and perinuclear region. Here, we expand the knowledge of graphene–cell interaction, focusing on the modulation of intracellular cell-death pathways. Additionally, we performed an in vitro time-dependent study to measure the initial responsiveness of tumour cells to chemotherapeutic drugs, and contextually evaluated the GCD@DOX-mediated changes in p53-related signalling. Although the extensive bibliography provided important information about the interactions between graphene-related nanomaterials and the induction of programmed cell death, reporting the impact of graphene on intracellular components, the production and secretion of proinflammatory cytokines, and the activation of a cascade of events [23,24,25,26,27,28,29,30], it should be considered inclusive with respect to differently functionalised graphene. In fact, the nanoplatform GCD, originated by engineering of G with CD, is endowed with unique properties originating from the synergic actions of single components that are not the result of the simple combination of the starting properties of native materials.

## 2. Materials and Methods

### 2.1. Synthesis of Drug-Loaded GCD

GCD@DOX complex was prepared using GCD and Doxorubicin (DOX) according to procedure previously reported. DOX content in GCD@DOX was 2.5% [18]. Thus, 25 μg/mL of GCD@DOX contains 0.625 μg/mL of DOX.

### 2.2. Cell Cultures

Cell lines were originally obtained from the American Type Culture Collection (ATCC). HEp-2 cells were grown in RPMI 1640 medium (Lonza, Belgium), supplemented with 10% FBS, 100 U/mL penicillin and 100 μg/mL streptomycin mixture, and were cultured at 37 °C in a 5% CO_2_ incubator.

### 2.3. Antibodies

Antibodies to p-Cdk (Thr14/Tyr15)-R (sc-28435-R) and to Wee-1 (B11; sc-5885) were purchased from Santa Cruz Biotechnology (Dallas, Texas, USA). Antibodies to phospho-p53 (Ser15; #9284), p21 Waf1/Cip1 (12D1; #2947), LC3B (D11; #3868), and SQSTM 1-p62 (D5L7G; #88588) were provided by Cell Signaling Technology^®^ (Danvers, MA, USA). Polyclonal antibodies against the housekeeping gene GAPDH and Histone H3 were purchased from Abcam (Cambridge, UK) and Cell Signaling Technology^®^, respectively. Secondary antibodies anti-rabbit and anti-mouse IgG conjugated to peroxidase were also from Merck Millipore (Darmstadt, Germany).

### 2.4. Protein Extractions and Immunoblot Analysis

To obtain total protein extraction, cells were lysed in 62.5 mM Tris-HCl pH 6.8; DTT 1 M; 10% glycerol; 2% SDS; 0.01% Bromophenol Blue and held at 100 °C for 5 min. Nuclear and cytoplasmic fractions were isolated as reported elsewhere [31] and analysed for protein determination using a Qubit™ Protein Assay Kit (Invitrogen™, Waltham, MA, USA). An equal amount of protein extracts was subjected to Sodium dodecyl sulfate-polyacrylamide gel electrophoresis (SDS-PAGE) and transferred to nitrocellulose membranes (Bio-Rad Life Science Research, Hercules, CA, USA). After incubation in blocking buffer, membranes were probed overnight at 4 °C with the specific primary antibody and then probed for 1 h at RT with secondary antibodies, followed by chemiluminescent detection, according to the manufacturer’s instructions. Protein bands were visualised by using Immobilon Classico Western HRP substrate (Merk, Millipore, Burlington, MA, USA) and captured using a ChemiDoc Touch Imaging System (Bio-Rad, Hercules, CA, USA), where indicated. Quantitative densitometry analysis of immunoblot band intensities was performed using ImageJ software and graphically represented using GraphPad Prism 6 software (GraphPad Software, San Diego, CA, USA). Statistical analysis was performed by ANOVA followed by Bonferroni’s Multiple Comparison Test.

### 2.5. Cell Viability Assay

The cell viability of HEp-2 cells treated with DOX, GCD and GCD@DOX was determined on the basis of ATP levels using ViaLightTM plus cell proliferation and cytotoxicity bioassay kit according to the manufacturer’s instructions (Lonza Group Ltd., Basel, Switzerland). Cells were grown in 96-well plates and treated with different concentrations of indicated DOX (1.25 μg/mL), GCD (25 μg/mL) and GCD@DOX (25 μg/mL). The contents of DOX in 25 μg/mL of GCD@DOX was 0.625 μg/mL. After the indicated incubation time, the cells were harvested and the emitted light intensity related to ATP degradation was quantified with the GloMax Multi Microplate Luminometer (Promega Corporation, 2800 Woods Hollow Road Madison, WI, USA). The luminescence value was converted to the cell proliferation index and reported as percentage of cell viability (%) according to the following equation:

Cell viability% = [(A−B)/(C−B)]% (1)

where A denotes the average of treated sample, B represents background luminescence, and C represents the average of untreated samples.

### 2.6. Acridine Orange Assay

The morphological analysis of apoptotic cells was performed following staining with the fluorescent DNA-binding dye acridine orange (AO). Thus, HEp-2 cells were untreated and treated with 1.25 μg/mL of DOX and 25 μg/mL of GCD@DOX for 24 h, 48 h and 72 h, collected on poly l-lysine-coated slides and stained with AO to determine the amount of apoptotic cells through fluorescence microscopy [32].

### 2.7. Evaluation of Autophagy by Tandem mRFP-GFP-LC3 and by LC3-I, LC3-II/SQSTM-p62 Autophagy-Related Protein Detection

Hep-2 cells were grown in multiwell culture slides and transiently transfected for 48 h with mRFP-GFPLC3 plasmid [33]. Twenty-four hours post-transfection, the cells were untreated and treated with 1.25 μg/mL and 25 μg/mL of DOX and GCD@DOX, respectively. Then, the samples were collected and washed twice with warm PBS, fixed with paraformaldehyde (PFA) 4% for 30 min at room temperature, and permeabilised with Triton 0.1% for 1 h, covered with a drop of mounting solution (ProLong Diamond Antifade Mountant with DAPI-Invitrogen p36971) for 30 min in a dark room. The images were captured and processed using confocal laser scanning microscopy TCS SP8, Leica, (TCS SP8, Leica Microsystems Srl, Milan, Italy) (magnification, 63×). For the expression analysis of LC3-I, LC3-II and SQSTM-p62 proteins, HEp-2 cells were untreated or treated with GCD (25 μg/mL), GCD@DOX (25 μg/mL) and DOX (1.25 μg/mL) for 24 h, 48 h and 72 h. The cells were then collected and processed for protein extraction and immunoblot analysis.

## 3. Results

### 3.1. In Vitro Evaluation of DOX, GCD and GCD@DOX Biocompatibility 

**Figure 1:**
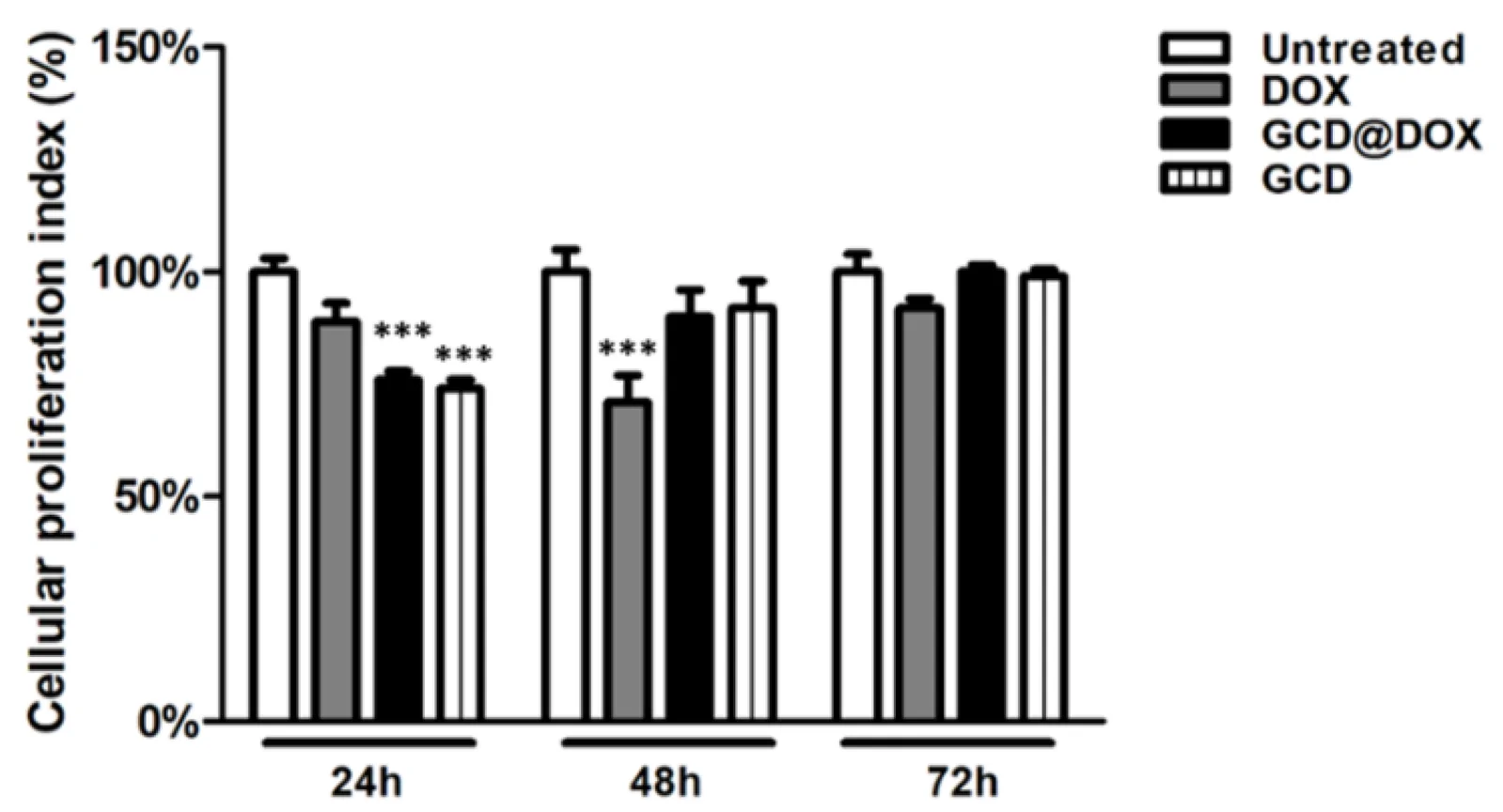
Time course study of biocompatibility of DOX (1.25 μg/mL), GCD (25 μg/mL) and GCD@DOX (25 μg/mL) in HEp-2 cells. The cells were untreated or treated with DOX, GCD or GCD@DOX for 24 h, 48 h and 72 h. The data show the % of live cells compared to the untreated ones. The cellular proliferation index (%) was determined on the basis of ATP level as described in Materials and Methods. GraphPad Prism 6 software was used for data analysis and graphical representation. The assay was performed as means of triplicates ±SD. Statistical significance was tested by one-way ANOVA analysis assay in triplicate. (*** *p* < 0.001).

Our recent findings regarding GCD indicated its ability to deliver DNA plasmid and miRNAs [17]. Moreover, we established that the fluorescence-labelled platform (GCD@Ada-Rhod) at a concentration of 25 µg/mL does not induce a cytotoxic effect and efficiently crosses cell membranes delivering genetic material. Herein, GCD was loaded with DOX to verify the drug-release efficiency by analysing specific intracellular signals. The sensibility and degree of resistance to DOX on HEp-2 cells was tested by monitoring the cytotoxicity at different treatment times (Figure 1). HEp-2 cells were pre-incubated overnight in 96-well plates with a final volume of 100 µL/well at 37 °C and 5% CO_2_ and treated for 24 h, 48 h and 72 h with 1.25 μg/mL of DOX and 25 μg/mL of GCD@DOX and GCD. The results showed a low degree of toxicity after GCD and GCD@DOX treatment at 24 h (24% and 26%, respectively) (*p* < 0.001), with a late recovery of vitality at 48 h and 72 h. Conversely, DOX treatment was moderately toxic (29%) at 48 h after exposure (*p* < 0.001).

### 3.2. Investigation of p53 and Wee-1 Signalling Mediated by DOX, GCD and GCD@DOX Treatment

**Figure 2:**
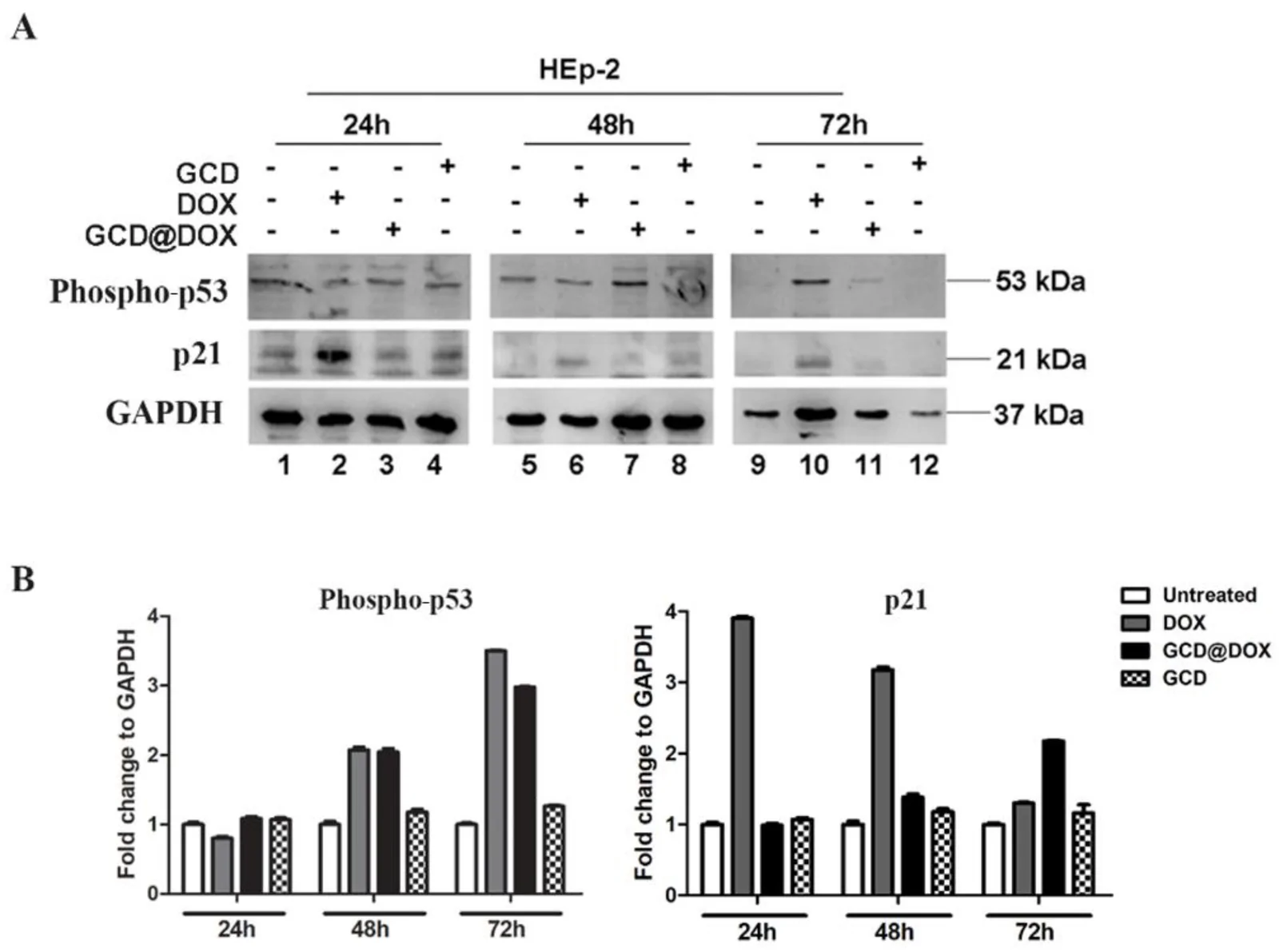
Phospho-p53 and p21 protein expression in GCD@DOX-, GCD- and DOX-treated cells. HEp-2 cells were treated or untreated with 25 μg/mL of GCD@DOX for the indicated time (24 h, 48 h and 72 h). DOX and GCD were used as controls. (**A**) An equal amount of proteins was separated by polyacrylamide gel electrophoresis and probed with a specific antibody to phospho-p53 and p21. GAPDH was used as housekeeping gene. (**B**) The quantitative densitometric analysis for phospho-p53 and p21 band intensities was determined using ImageJ software and expressed as fold change over the appropriate housekeeping gene.

The signal transduction pathways in response to damaged DNA lead to programmed cell death, repair mechanisms, or cell cycle arrest [34,35,36]. Ordinarily, checkpoint signalling ensures that DNA replication is arrested in damaged cells through proteins stalling the cell cycle or activating apoptotic mechanisms. The tumour-suppressor protein p53 is induced by DNA damage signals and counteracts the genomic instability of cancer cells through a wide range of biological processes [37,38]. Thus, the increased p53 expression promotes the accumulation of p21 (also known as p21 WAF1/Cip1), which is responsible for growth arrest through inhibition of the cyclin/cyclin-dependent kinase (CDK) complex, required for G1/S transition [39,40]. Therefore, HEp-2 cells were untreated and treated with 1.25 μg/mL of DOX and 25 μg/mL of GCD@DOX and GCD and collected after 24 h, 48 h, and 72 h of treatment. The accumulation of phospho-p53 and p21 was detected by Western blot analysis. As shown in Figure 2A and graphically reported in Figure 2B, an accumulation of phospho-p53 was detected in free DOX- and GCD@DOX-treated cells at 48 h and 72 h (Figure 2A, lanes 6, 7, 10 and 11). The expression of p21 peaked at 24 h and 48 h following free DOX treatment (Figure 2A lanes 2 and 6) and decreased over time. Conversely, p21 levels slowly increased at 72 h following GCD@DOX treatment (Figure 2A lanes 11 and Figure 2B). No significant changes in phospho-p53 or p21 expression levels were detected following treatment with GCD compared to untreated cells. Thus, the GCD alone did not impact the p53/p21 signalling.

**Figure 3:**
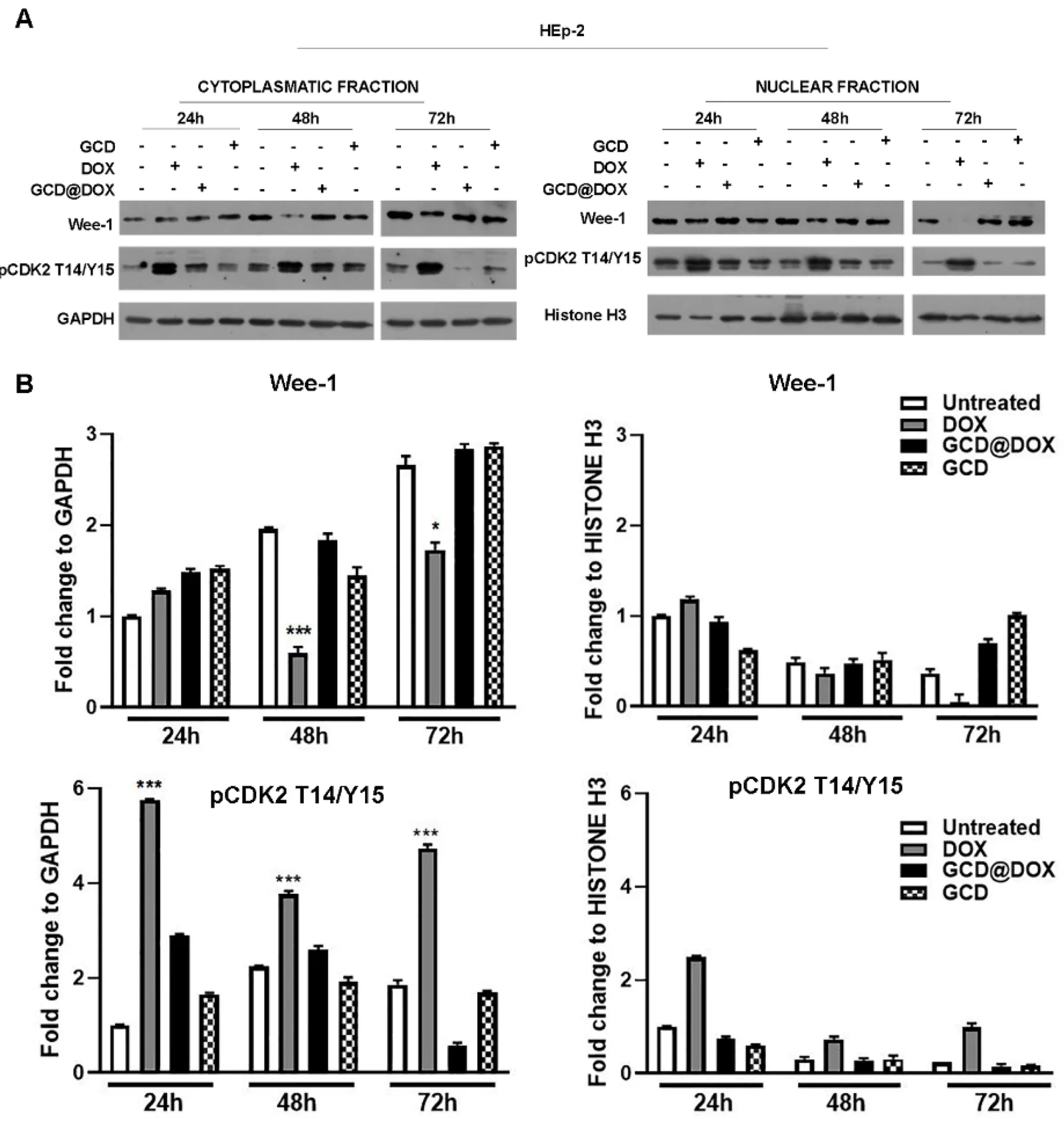
Wee-1 and phospho-CDK2 (pCDK2 T14/Y15) protein expression in GCD@DOX-, GCD- and DOX-treated cells. HEp-2 cells were untreated or untreated with 25 μg/mL of GCD@DOX for 24 h, 48 h and 72 h. DOX (1.25 μg/mL) and GCD (25 μg/mL) were used as controls. (**A**) An equal amount of cytoplasmic and nuclear proteins was separated by polyacrylamide gel electrophoresis and probed with Wee-1 and phospho-CDK2 (pCDK2 T14/Y15) antibodies. GAPDH and Histone H3 were used as a loading control for the cytoplasmic and nuclear fractions, respectively. (**B**) The quantitative densitometric analysis of Wee-1 and pCDK2 T14/Y15 band intensities was determined in the cytoplasmatic and nuclear fractions for both with ImageJ software and expressed as fold change over the appropriate housekeeping gene. (* *p* < 0.1; *** *p* < 0.001).

While p53 and p21 are considered mediators of G_0_/G_1_ cell cycle arrest, Wee-1 blocks G_2_/M transition [41]. Wee-1 is an inhibitory kinase that catalyses the phosphorylation of tyrosine 15 (Y15) on CDK, rendering it inactive and blocking CDK-cyclin interaction and cell cycle progression [41,42]. Then, the spatio-temporal regulation of Wee-1 was detected on HEp-2 cell lines, untreated and treated with DOX (1.25 μg/mL), GCD@DOX 25 μg/mL and GCD (25 μg/mL), and collected at 24 h, 48 h, and 72 h of treatment. The results are shown in Figure 3A and graphically reported in Figure 3B. We found that in both the untreated cells and cells treated with GCD and GCD@DOX, Wee-1 expression grew over time in the cytoplasm and maintained unchanged levels in the nucleus. Conversely, Wee-1 accumulation was significantly inhibited by DOX treatment in both cellular compartments (* *p* < 0.1; *** *p* < 0.001) compared with the untreated cells. At the same time, DOX influences pCDK2 T14/Y15; indeed, increased levels were detected in the cytoplasm and nucleus compared to untreated cells (*** *p* < 0.001). 

### 3.3. Programmed Cell Death in HEp-2 Cells following DOX, GCD and GCD@DOX Treatment

**Figure 4:**
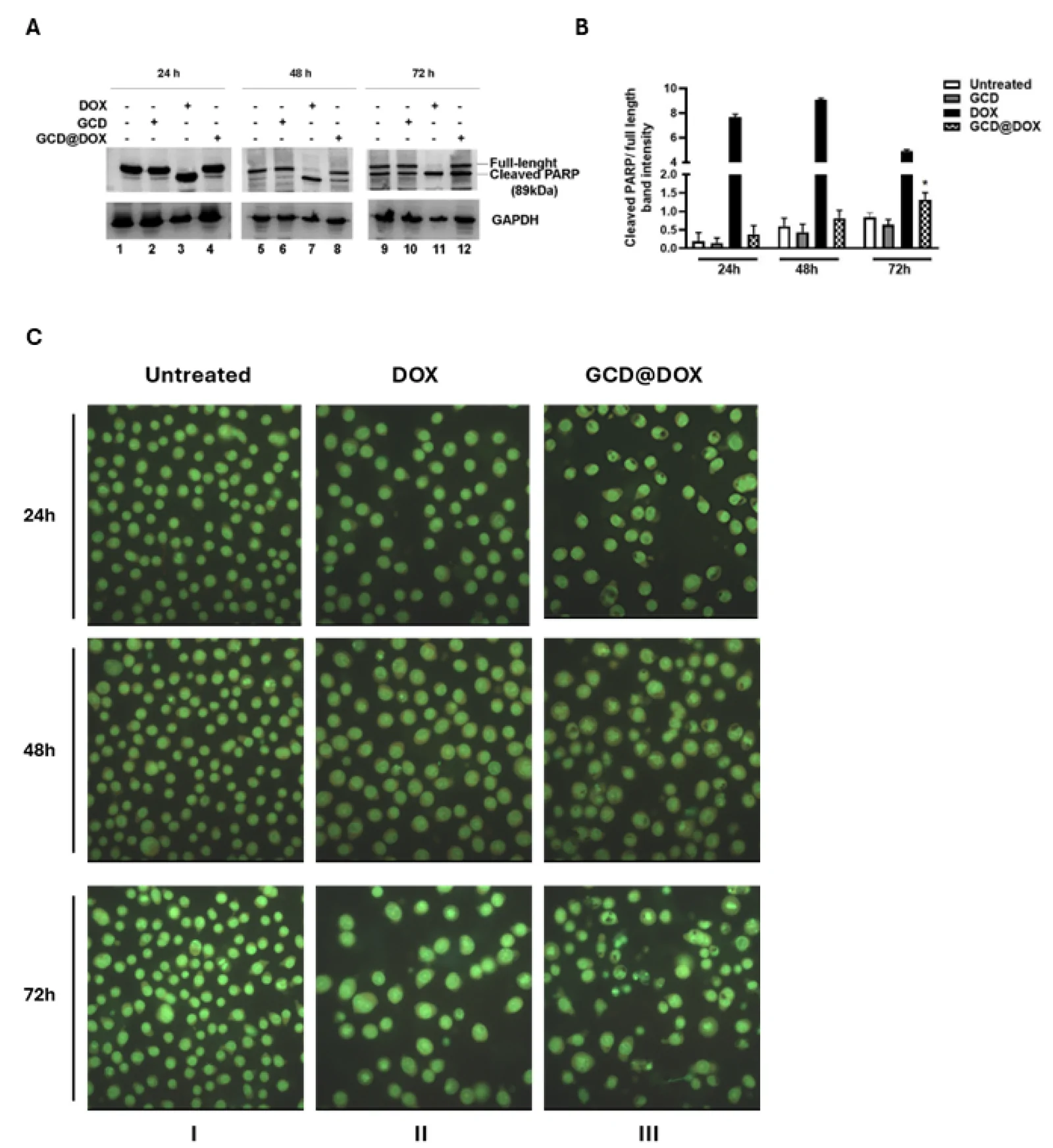
Monitoring of apoptosis in HEp-2 cells following DOX, GCD and GCD@DOX treatment. HEp-2 cells were untreated and treated with 1.25 μg/mL of DOX and 25 μg/mL of GCD@DOX and GCD and collected at 24 h, 48 h and 72 h post-treatment. (**A**,**B**) The Western blot analysis was performed to detect PARP protein expression. The protein bands were visualised by using Immobilon Classico Western HRP substrate (Merk, Millipore) and captured using a ChemiDoc Touch Imaging System (Bio-Rad). The quantitative densitometry analysis of cleaved PARP/full length band intensity was performed by using ImageJ software and is graphically represented in (**B**) using GraphPad Prism 6 software (GraphPad Software, San Diego, CA, USA). * *p* < 0.05, compared with the GCD treated cells. (**C**) The morphological analysis of apoptotic cells was performed following staining with the fluorescent DNA-binding dyes acridine orange, at 24 h, 48 h and 72 h post-treatment (I: untreated; II: DOX; III: GCD@DOX) and visualised by fluorescence microscope (Leitz, Wetzlar, Germany) (magnification, 63×).

The apoptotic process represents a programmed cell death (PCD) mechanism that normally provides homeostatic maintenance and cell survival [43]. An unbalanced apoptotic activity is associated with the onset of injuries and several pathologies. In cancer disease, low levels of apoptosis provoke an accumulation of malignant cells and also depend on faulty cellular pathways [44,45,46]. The activation of apoptosis is characterised by specific biochemical and morphological changes such as apoptotic body formation, chromatin condensation and nuclear fragmentation. First of all, we studied the apoptotic process by analysing the cleavage of poly(ADP-ribose) polymerase-1 (PARP), following antitumour drug intracellular delivery [47]. HEp-2 cells were untreated or treated with 25 μg/mL of GCD@DOX for 24 h, 48 h and 72 h. Free DOX (1.25 μg/mL) and GCD (25 μg/mL) were used as controls. The cleaved PARP-1 expression is shown in Figure 4A and graphically reported in Figure 4B. The Western blot analysis reported the cleavage of PARP following treatment with free DOX starting from 24 h post-exposure (Figure 4A lanes 3, 7, 11). Conversely, GCD@DOX treatment led to an accumulation of cleaved PARP at 72 h (*p* < 0.05) (Figure 4A lane 12) indicating a slow release of the drug by the carrier. No significant differences were observed between the GCD-treated and untreated cells, suggesting that the graphene carrier does not affect the cleavage of PARP. In addition, we observed the morphological changes related to apoptotic activation. The cells were first stained with acridine orange (AO), which permeates all cells and makes the nuclei appear green, and then observed on a fluorescence microscope detecting the green emission [48]. The acridine orange-negative cells with a normal green nucleus and homogeneous chromatin distribution were detected in untreated samples (Figure 4C(I)). A limited number of cells with condensed or fragmented chromatin in the green nucleus, debris particles and formation of apoptotic bodies were observed only after GCD@DOX exposure (Figure 4C(III)). DOX treatment led to rounded cellular phenotype 72 h post-exposure not strictly related to typical morphological features of apoptosis (Figure 4C(II)).

### 3.4. Monitoring of Autophagy following GCD@DOX and DOX Treatment

**Figure 5:**
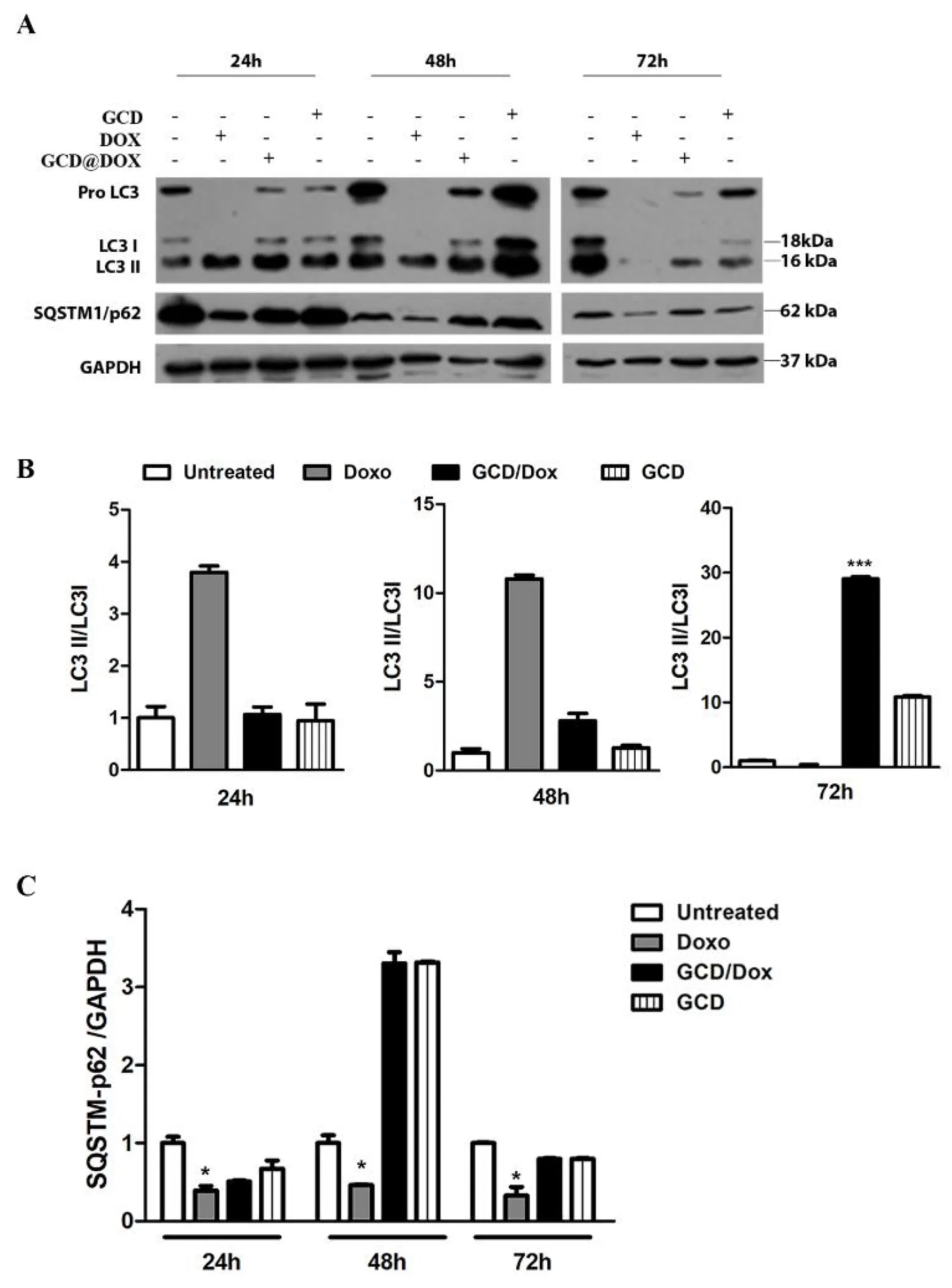
Parallel monitoring of LC3-I, LC3-II and SQSTM-p62 autophagy-related protein expression in HEp-2 cells treated with GCD@DOX and DOX. (**A**) Immunoblot and quantification of LC3-I, LC3-II and SQSTM-p62 in HEp-2 cells untreated or treated with GCD@DOX, DOX and GCD for the indicated times. (**B**,**C**) The graphs show (mean ± SD) LC3-II/LC3-I ratios of each treatment normalised to the LC3-II/LC3-I ratio of control-untreated cells for each experiment. Statistical significance was tested by one-way ANOVA analysis assay in triplicate. *** *p* < 0.001 and * *p* < 0.05 indicate significant changes vs. control.

**Figure 6:**
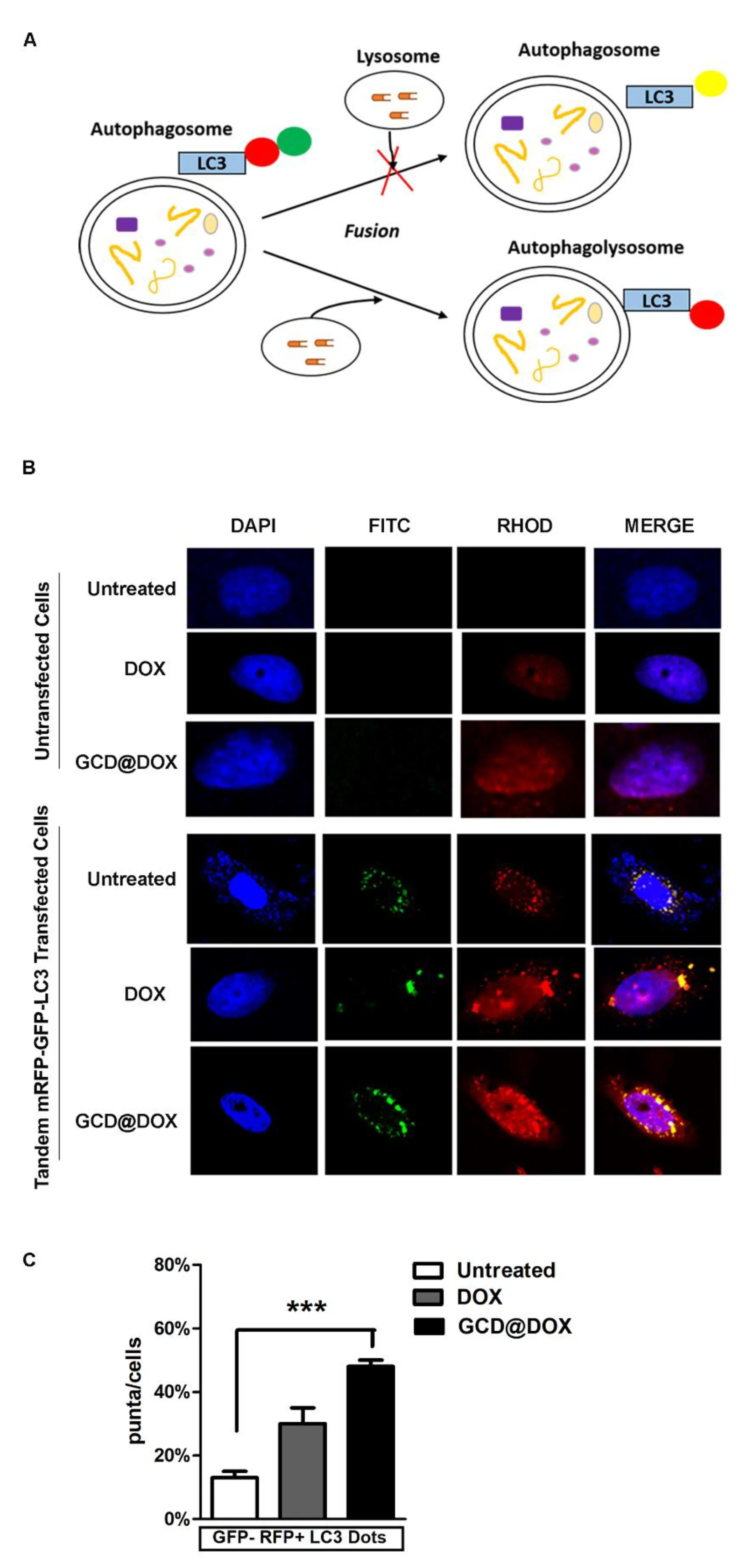
Monitoring of the double-tagged LC3 by mRFP-GFP-LC3 tandem fluorescent protein. (**A**) Graphical representation of mRFP-GFP-LC3 tandem. (**B**) Representative confocal images of HEp-2 cells untransfected and transiently transfected with mRFP-GFP tandem fluorescent-tagged LC3 and untreated or treated with DOX (1.25 μg/mL) and GCD@DOX (25 μg/mL) for 48 h (magnification, 63X). (**C**) Quantification of GFP^−^/RFP^+^/LC3 dots represented as percentage (%) in HEp-2 untreated and treated with DOX and GCD@DOX. Statistical significance was tested by one-way ANOVA analysis assay in triplicate (*** *p* < 0.001).

In general, anti-cancer therapy promotes the simultaneous activation of different death signals in order to beat heterogeneous tumour cell populations. In particular, the autophagic mechanism degrades damaged cellular components, recycles cellular organelles, and protects cells from several types of stress, maintaining genomic integrity [49]. However, it is well known that autophagy plays a dual role in cancer development, as either a tumour suppressor for inhibiting tumour progression, or as a cell survival mechanism for promoting tumour growth. For example, increased autophagy and diminished apoptosis were found in DOX-resistant multiple myeloma RPMI8226/DOX cells. Indeed, Pan and collaborators demonstrated that autophagy has a protective role in cancer cells, which results in decreased sensitivity to DOX [50]. To investigate the autophagy process following GCD@DOX exposure, we monitored autophagosomes formation using a genetic approach and quantified the expression of key regulators of autophagy. Autophagy is a dynamic multistep process, regulated by more than 30 autophagy proteins (ATGs), which consists of the formation of autophagosomes, the fusion of the autophagosome with the lysosome to form the autolysosome, and the degradation of the contents in the autolysosome [51,52,53]. During this process, microtubule-associated protein 1A/1B light chain 3 (LC3-I) is generated by proteolytic cleavage of pro-LC3 and conjugated to phosphatidylethanolamine (PE) to form LC3-II. LC3-II is recruited and incorporated onto the autophagosomal membrane and interacts with the Sequestosome1/p62 (SQSTM1) protein. The accumulation of LC3-II is correlated with autophagosome synthesis and, together with the monitoring of p62 degradation, describes the ongoing autophagosome maturation process [33,54,55]. The analysis of the expression levels of LC3-II detected through immunoblot (Figure 5) showed distinct bands for pro-LC3, LC3-I and LC3-II at 24 h, 48 h and 72 h in untreated cells (Figure 5 lanes 1,5,8). The LC3-II/LC3-I ratio was calculated on the basis of a densitometric analysis of both bands and is presented in a simplified form in Figure 5B. The results show an accumulation of LC3-II/LC3-I after 24 h and 48 h of DOX exposure (Figure 5A,B) and low p62 levels at all considered times (Figure 5C). Conversely, high levels of LC3-II/LC3-I and p62 were detected after GCD@DOX treatment at 72 h and 48 h, respectively. Any significant effect on the LC3-II accumulation occurred as a result of GCD exposure over time, or was otherwise due to increased p62 levels. The results showed an increase in the LC3-II pool in comparison to LC3-I by DOX exposure as a sign of autophagy induction and a reduction of p62 levels. A high level of LC3-II was belatedly (72 h) detected after GCD@DOX treatment in a GCD-independent manner and without tending to p62 degradation. This indicated that GCD@DOX exposure was correlated with an increased number of autophagosomes, indicating the activation of autophagy. In order to track different stages of autophagy and verify whether the autophagosome maturation process evolved into endosome or lysosome fusion, the mRFP-GFP tandem fluorescent-tagged LC3 assay was employed. The double-tagged LC3B was used to differentially label the autophagosomes and autolysosomes due to the different pH stability of both probes. In fact, differential quenching of the fluorescence was emitted by the red (mRFP) and green (GFP) proteins in the lysosomal acidic compartment [56]. The graphical representation of mRFP-GFP tandem fluorescent-tagged LC3 is shown in Figure 6A. Briefly, autophagosomes appear as yellow dots (GFP^+^/RFP^+^), because both tags emit overlapping fluorescent light. Conversely, the autolysosomes are red dots (GFP^−^/RFP^+^), because the GFP fluorescence, unstable in the acidic lysosome environment, is lost. Therefore, HEp-2 cells were transiently transfected for 48 h with mRFP-GFPLC3 plasmid and 24 h after transfection were untreated and treated with 1.25 μg/mL of DOX and 25 μg/mL of GCD@DOX, collected, and analysed with confocal microscopy. DOX has an intrinsic bright fluorescence that diffuses into the cells and an emission signal at 595nm. The results in Figure 6B,C show that: (i) in untreated cells, the number of green and red dots overlaps and represents the basal autophagy level. (ii) In DOX-treated cells, about 30% of red dots were detected. (iii) In GCD@DOX-treated cells, about 50% of the red dots were detected. These findings suggest that the exposure to DOX and GCD@DOX significantly triggers the clear accumulation of autolysosomes, GFP-RFP + LC3, and indicates the activation of the autophagy process.

## 4. Discussion

Many studies have recently pointed out the efficiency of DOX loaded into nanoparticles, which is able to overcome the limitations of conventional treatments in cancer therapy [57,58]. DOX exerts an anti-cancer effect through intercalation into the DNA, inducing single- and double-strand breaks in the DNA [59]. Additionally, it interacts specifically with the inner mitochondrial membrane, leading to the production of reactive oxygen species (ROS) [60]. To date, chemotherapeutic treatment with DOX is associated with undesirable multi-organ toxicities, including liver, cardiac, neuronal, and muscle toxicity, hepatorenal profile alterations, and systemic inflammation [61,62]. Thus, its clinical uses have been restricted to low dosages. Therefore, DOX-based nanoformulations have been studied for their anticancer effect and for their ability to evade chemoresistance and DOX toxicity. Indeed, Andreopoulou and collaborators found lower values of cardiac toxicity in therapy with liposomal DOX plus Trastuzumab for metastatic breast cancer [63]. Modification of DOX with ligands, lipid–polymer hybrid nanoparticles, and liposomes is anticipated to improve the transport of DOX into tumour cells, as well as limiting its toxicity and activating anti-cancer intracellular signalling [64,65,66]. Recently, our group examined the stability and biological behaviour of an in vitro system of GCD@DOX on the murine C26 cell line and on the human HEp-2 cell line [18]. Changes in the expression of some genes associated with angiogenesis, such as extracellular matrix modification (ECM) and tumour metastasis, were found. In particular, GCD@DOX induced a strong down-regulation of genes that can be involved in tumorigenesis, including ST14, PLAUR, SP1, SF3A3, ITGB2, GSN, BMP5, TNFSF12, and AKT1. Otherwise, GCD alone affects four genes with an opposite trend compared to DOX loaded on GCD (VIM, TNFSF12, BICC1, SRPK2). Graphene affects cell biology in a variety of modes, and some effects can be beneficial; for example, increased VIM expression is correlated with resistance and poor outcome treatment in patients with different tumours. The detrimental increase in VIM expression induced by DOX could be softened by GCD-induced VIM down-regulation. This study provides further insights into the intracellular targets of DOX, which are involved in the clearance of tumour cells. In particular, we characterised graphene-mediated signalling and its role in DOX release, analysing the expression of p53 protein and the p53-related pathways. p53 plays a crucial role in supporting DNA repair by arresting the cell cycle, regulating apoptosis, and controlling autophagy [67]. Therefore, we reported that DOX and GCD@DOX induce p53 accumulation (Figure 2). The p53-dependent upregulation of the cell cycle inhibitor p21 occurs belatedly following GCD@DOX compared to the free DOX treatment, suggesting that GCD controls DOX release over time. While p53 and p21 are considered mediators of G0/G1 cell cycle arrest, Wee-1 blocks G2/M transition. Wee-1 is an inhibitory kinase that catalyses the phosphorylation of tyrosine 15 (Y15) on CDK, rendering it inactive and blocking CDK–cyclin interaction and cell cycle progression [41,42]. We found that DOX treatment triggers the reduction of Wee-1 protein expression, unlike GCD and GCD@DOX, whose levels are comparable over time to endogenous expression (Figure 3). The combined decrease of Wee-1 and accumulation of pCDK (T14/Y15) by DOX is consistent with the inhibition of Wee-1 in cancer treatment reported in previous studies [41]. Elbæk and collaborators reported that CDK drives the proteasomal degradation of Wee-1, leading to its decrease. Additionally, the inhibition of Wee-1 overrides the cell cycle arrest and results in mitotic entry [42]. This fact could explain the genotoxic behaviour induced by chemotherapeutic drugs, and emphasises the importance of nanotechnology in drug delivery. Additionally, p53 can promote non-apoptotic cell death via p53-PARP crosstalk [68]. The PARP activation can be attributed to ROS generation induced by cytotoxic drugs [69]. Our findings report a cleavage of PARP by DOX within 72 h of treatment. Unlike, GCD@DOX induces a late cleavage of PARP, which can be indicative of a less toxic effect as a result of controlled drug release (Figure 4). Additionally, the existence of the p21/PARP-1 axis has been reported to be involved in DNA damage and repair [70]. In particular, p21 is sequestered by PARP-1, facilitating DNA repair mechanisms [71]. For this reason, the PARP-1 cleavage after DOX exposure, as shown in Figure 4A, does not match perfectly with the apoptosis induction reported through acridine orange staining (Figure 4C). Chromatin condensation, debris particles, and formation of apoptotic bodies, hallmarks of apoptosis, were poorly detected after GCD and GCD@DOX treatment and were potentially related to mechanical stress induced by graphene uptake, as indicated by the low toxicity levels detected at 24 h (Figure 1). In agreement with the literature, our data showed a cytotoxic effect of DOX after 48 h of exposure and a late recovery at 72 h, which was potentially linked to drug metabolism [72]. Conversely, GCD and GCD@DOX induced early weak toxicity at 24 h, but no considerable effect was detected over time. This phenomenon underlines the temporal biocompatibility of the nano-platform. Additionally, these differences in cytotoxicity could be related to the accumulation and efflux of the drug or its metabolites following free or graphene-mediated delivery. Together with the role of p53 in inducing apoptosis and growth arrest, a non-canonical p53 function has been reported [73,74]. Duan and collaborators reported that p53 activates the autophagy process through the inhibition of mTOR [73]. Autophagy is a catabolic process needed to maintain homeostasis and survival through the removal and recycling of unwanted cellular materials. Although autophagy, unlike apoptosis, which is a clear mechanism of cell death, represents a cellular pro-survival pathway [74], different studies have identified them as independent processes with interconnected cell death mechanisms [24,75,76,77]. Autophagy is characterised by the formation of double membrane-bound structures wrapping organelles, known as autophagosomes, which fuse with lysosomes to form autolysosomes and degrade the absorbed materials [52]. A set of established criteria is necessary to observe and validate autophagy induction. The localisation of LC3-II on the cytosolic surface of autophagosomes is widely used as a marker of autophagy [53,54,55]. Here, the clear accumulation of autolysosomes, GFP-RFP + LC3, was reported after exposure to DOX and GCD@DOX. This increase, observed through tandem fluorescent-tagged LC3 (mRFP-EGFP-LC3) assay, was statistically significant (Figure 6). In parallel with monitoring LC3-II, tracking the conversion of LC3-I to LC3-II and the degradation of p62, which are factors indicative of autophagic activity, are necessary for monitoring autophagic flux [55,78]. We showed an increase in the LC3-II pool in comparison to LC3-I with DOX exposure, along with a reduction in p62 levels (Figure 5). Conversely, GCD@DOX treatment accumulates LC3-II belatedly, without degrading p62, and in a graphene-independent manner. Recent studies have demonstrated that autophagy induced by chemotherapeutic drugs may promote the resistance of cancer cells to drugs, together with decreased apoptosis [50,79]. Our results indicate that DOX treatment activates the autophagy flux and maintains it over time. This finding, combined with the cell cycle regulation reported previously, may support the hypothesis that a combined effect of both processes could be useful for limiting the existence and persistence of heterogeneous tumour cell populations. Otherwise, GCD@DOX activates autophagy, but rather than promoting the immediate degradation of cellular substrates useful for tumour growth, it supports the recycling of DOX, limiting its toxicity. Indeed, because the drugs used in chemotherapy result in highly deleterious and often life-threatening side effects, a controlled drug delivery system, mediated by a graphene-based platform, could target sub-cellular sites, reducing cytotoxicity.

## 5. Conclusions

Overall, the present investigation allowed us to characterise some of the cellular pathways commonly recruited in nanomaterial treatments such as autophagy. Our results showed that the intracellular delivery of DOX mediated by the graphene platform GCD activates the p53 pathway, induces apoptosis signalling, and sustains the removal and recycling of DOX by activation of autophagy. The developed graphene-based drug delivery tool could offer an approach to the intracellular delivery of DOX, maximising its therapeutic effect and limiting its toxicity. Nevertheless, a deeper understanding of GCD-autophagy interaction at the mechanistic and functional level is needed before these findings can be exploited to increase the effectiveness of GCD in cancer therapeutics and drug delivery.

## Data Availability

Not applicable.
