# Correction: Pennisi et al. Cancer-Related Intracellular Signalling Pathways Activated by DOXorubicin/Cyclodextrin-Graphene-Based Nanomaterials. *Biomolecules* 2022, *12*, 63

**DOI:** 10.3390/biom16081134

**Published:** 2026-08-04

**Authors:** Rosamaria Pennisi, Maria Musarra-Pizzo, Tania Velletri, Antonino Mazzaglia, Giulia Neri, Angela Scala, Anna Piperno, Maria Teresa Sciortino

**Affiliations:** 1 Department of Chemical, Biological, Pharmaceutical and Environmental Sciences, University of Messina, 98166 Messina, Italy; mmusarrapizzo@unime.it (M.M.-P.); giulia.neri@unime.it (G.N.); ascala@unime.it (A.S.); apiperno@unime.it (A.P.); 2 IFOM-Cogentech Società Benefit srl; via Adamello 16, 20139 Milan, Italy; tania.velletri@cogentech.it; 3 Istituto per lo Studio dei Materiali Nanostrutturati, Consiglio Nazionale delle Ricerche (ISMN-CNR), V.le F. Stagno d’Alcontres 31, 98166 Messina, Italy; antonino.mazzaglia@cnr.it

In the original publication [1], there was a mistake in Figure 4C as published. Specifically, the image corresponding to the untreated condition at 72 h was inadvertently duplicated from the 24 h untreated condition, resulting in an incorrect representation of the 72 h control panel. This was a figure preparation error only and did not involve any manipulation or alteration of experimental data. The correct image for the untreated 72 h condition has now been inserted in the revised version of Figure 4C. The authors state that the scientific conclusions are unaffected. This correction was approved by the Academic Editor. The original publication has also been updated.

## Figures and Tables

**Figure 4:**
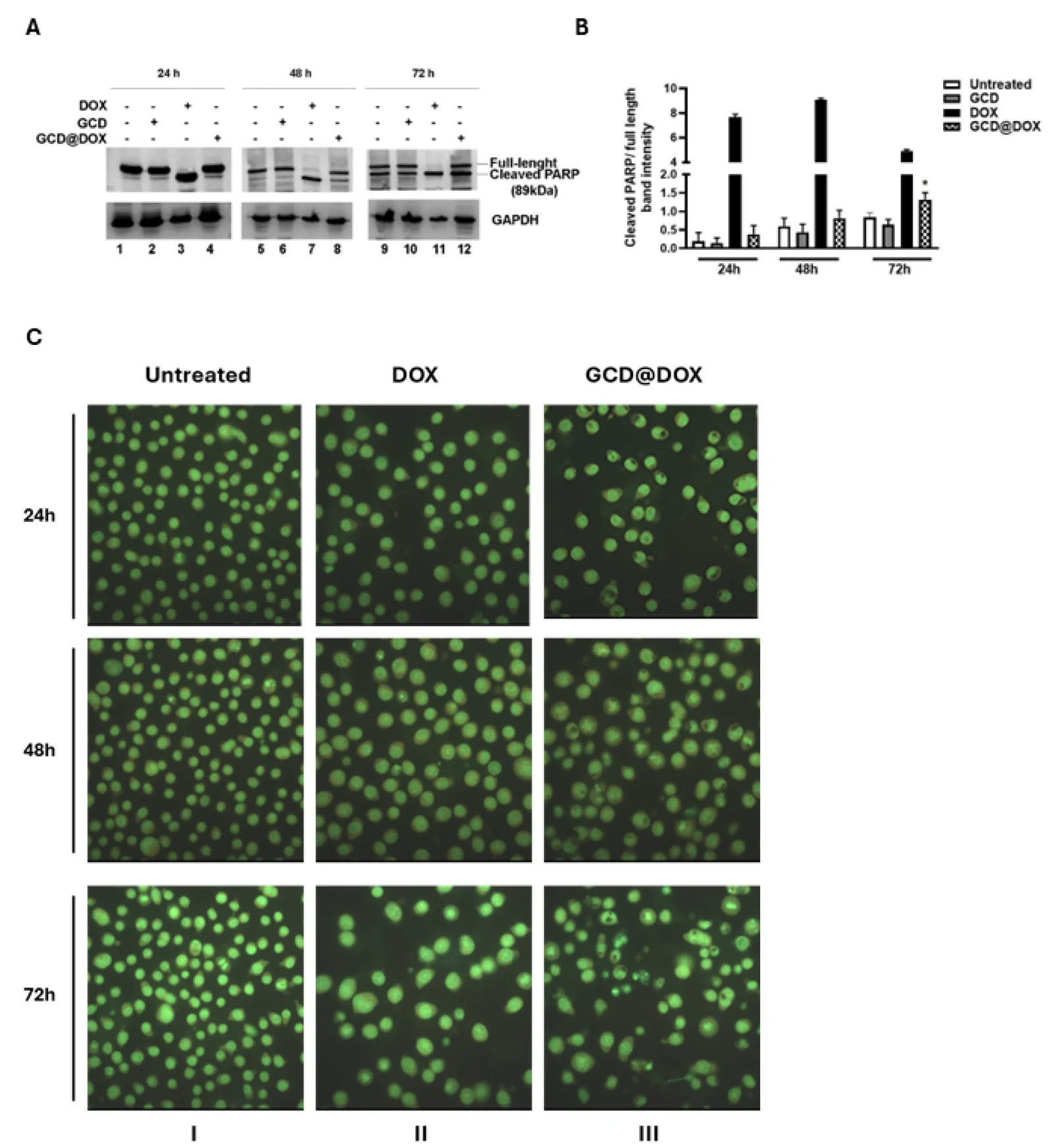
Monitoring of apoptosis in HEp-2 cells following DOX, GCD and GCD@DOX treatment. HEp-2 cells were untreated and treated with 1.25 μg/mL of DOX and 25 μg/mL of GCD@DOX and GCD and collected at 24 h, 48 h and 72 h post-treatment. (**A**,**B**) The Western blot analysis was performed to detect PARP protein expression. The protein bands were visualised by using Immobilon Classico Western HRP substrate (Merk, Millipore) and captured using a ChemiDoc Touch Imaging System (Bio-Rad). The quantitative densitometry analysis of cleaved PARP/full length band intensity was performed by using ImageJ software and is graphically represented in (**B**) using GraphPad Prism 6 software (GraphPad Software, San Diego, CA, USA). * *p* < 0.05, compared with the GCD treated cells. (**C**) The morphological analysis of apoptotic cells was performed following staining with the fluorescent DNA-binding dyes acridine orange, at 24 h, 48 h and 72 h post-treatment (I: untreated; II: DOX; III: GCD@DOX) and visualised by fluorescence microscope (Leitz, Wetzlar, Germany) (magnification, 63×).
